# Absolute and relative quantitation of amylase/trypsin-inhibitors by LC-MS/MS from wheat lines obtained by CRISPR-Cas9 and RNAi

**DOI:** 10.3389/fpls.2022.974881

**Published:** 2022-08-29

**Authors:** Sabrina Geisslitz, Shahidul Islam, Lukas Buck, Clemens Grunwald-Gruber, Francesco Sestili, Francesco Camerlengo, Stefania Masci, Stefano D’Amico

**Affiliations:** ^1^Department of Bioactive and Functional Food Chemistry, Institute of Applied Biosciences, Karlsruhe Institute of Technology, Karlsruhe, Germany; ^2^Leibniz Institute for Food Systems Biology at the Technical University of Munich, Freising, Germany; ^3^Department of Plant Sciences, North Dakota State University, Fargo, ND, United States; ^4^Core Facility Mass Spectrometry, BOKU - University of Natural Resources and Life Sciences, Vienna, Austria; ^5^Department of Agricultural and Forest Sciences, University of Tuscia, Viterbo, Italy; ^6^Austrian Agency for Health and Food Safety, Institute for Animal Nutrition and Feed, Vienna, Austria

**Keywords:** non-celiac wheat sensitivity, reduced immunogenic potential, iTRAQ, iBAQ, data independent acquisition, data dependent acquisition, durum wheat, common wheat

## Abstract

Quantitation of wheat proteins is still a challenge, especially regarding amylase/trypsin-inhibitors (ATIs). A selection of ATIs was silenced in the common wheat cultivar Bobwhite and durum wheat cultivar Svevo by RNAi and gene editing, respectively, in order to reduce the amounts of ATIs. The controls and silenced lines were analyzed after digestion to peptides by LC-MS/MS with different approaches to evaluate changes in composition of ATIs. First, a targeted method with stable isotope dilution assay (SIDA) using labeled peptides as internal standards was applied. Additionally, four different approaches for relative quantitation were conducted, in detail, iTRAQ labeled and label free quantitation (LFQ) combined with data dependent acquisition (DDA) and data independent acquisition (DIA). Quantitation was performed manually (Skyline and MASCOT) and with different proteomics software tools (PLGS, MaxQuant, and PEAKS X Pro). To characterize the wheat proteins on protein level, complementary techniques as high-performance liquid chromatography (HPLC) and gel electrophoresis were performed. The targeted approach with SIDA was able to quantitate all ATIs, even at low levels, but an optimized extraction is necessary. The labeled iTRAQ approach revealed an indistinct performance. LFQ with low resolution equipment (IonTrap) showed similar results for major ATIs, but low abundance ATIs as CM1, were not detectable. DDA measurements with an Orbitrap system and evaluation using MaxQuant showed that the relative quantitation was dependent on the wheat species. The combination of manual curation of the MaxQuant search with Skyline revealed a very good performance. The DIA approach with analytical flow found similar results compared to absolute quantitation except for some minor ATIs, which were not detected. Comparison of applied methods revealed that peptide selection is a crucial step for protein quantitation. Wheat proteomics faces challenges due to the high genetic complexity, the close relationship to other cereals and the incomplete, redundant protein database requiring sensitive, precise and accurate LC-MS/MS methods.

## Introduction

Non-celiac wheat sensitivity (NCWS) has been proposed as a non-specific and non-IgE mediated type of a gut associated disorder. The triggers of NCWS are still under discussion, but the metabolic proteins of wheat, in particular amylase/trypsin-inhibitors (ATIs) seem to be the predominant activator of this gastrointestinal and partly neurological disease (Zevallos et al., 2017; Geisslitz et al., 2021). It has been shown that ATIs activate the toll-like receptor 4 TLR4-MD2-CD14 complex causing secretion of proinflammatory chemokines and cytokines, which results in the activation of the innate immunity and in symptoms typical for NCWS (Zevallos et al., 2017). Due to the overlapping symptoms of irritable bowel syndrome and NCWS, and due to the lack of specific serological markers for NCWS, the diagnosis and the search for the cause of gastrointestinal and extraintestinal symptoms are challenging. Furthermore, ATIs are the triggers of Bakers’ asthma, a wheat allergy, which affects up to 10% of flour workers (Salcedo et al., 2011; Huebener et al., 2015). Recent, studies indicated that ATIs might even have the potential to cause celiac disease (Huebener et al., 2015; Yu et al., 2022) or are recognized by gluten-detecting enzyme-linked immunosorbent assays (Fraberger et al., 2020). Nevertheless, the role of ATIs in celiac disease is scarcely investigated so far and occurrence of them in gluten (isolates) (Wieser and Scherf, 2018; Lexhaller et al., 2019) even increases the complexity of this question.

ATIs are part of the albumins/globulins and correspond to about 2–6% of wheat proteins and to about 30% of albumins/globulins (Altenbach et al., 2011; Geisslitz et al., 2018, 2020). As the name indicates, they are inhibitors of heterologous amylase and trypsin that play an important protective role against those enzymes from insects, mite, and mammals (van Loon et al., 2006; Priya et al., 2013). The ATI family includes different subunits, with about 150 amino acid residues resulting in molecular weights of 13.0–15.5 kDa. ATI subunits share the commonality that they have nine to ten cysteine residues that form four to five intrachain disulfide bonds, responsible for the resistance to heat and enzymatic degradation (Buonocore et al., 1977; Silano and Zahnley, 1978; Warchalewski, 1987). The subunits can be subdivided into monomeric (0.28), dimeric (0.19 and 0.53), tetrameric–also named chloroform/methanol (CM)-soluble–proteins including CM1, CM2, CM3, CM16, and CM17 and minor ATIs (CMX1/2/3, WASI, WTI, and WCI) (Altenbach et al., 2011; Geisslitz et al., 2020). All before mentioned ATIs have evidence on protein level in the UniProtKB database (The UniProt Consortium, 2017).

Generally, mass spectrometry (MS) is a very sensitive and selective analytical method. Depending on the scientific question or required sensitivity, peptides and the corresponding proteins are detected by proteomic-based methods using global untargeted (also called discovery proteomics) or targeted approaches. The accuracy and quality of peptide and protein identification are strongly affected by genomic and proteome sequence databases. In particular, wheat proteomics faces with challenges, because of incomplete and duplicated sequences within the database. For example, the UniProtKB database lists 274 proteins (15 reviewed and 259 unreviewed) when searching for “amylase trypsin inhibitor wheat” (query on April 8, 2022). Beside proteins from common wheat (*Triticum aestivum* L.) others from different wheat species such as wild emmer (*T. dicoccoides*), durum (*T. turgidum* subsp. *durum*), or diploid wheat species (e.g., *Aegilops tauschii*) are included. Clustering by 100% identity revealed that different entries contain identical amino acid sequences, e.g., the cluster ID of UniRef100_A0A077RSX3 (Cluster: Dimeric alpha amylase inhibitor) shows eleven entries from different species. Hence, the sequences of the isoforms being studied may often not be unique, which limits its clear identification and thus, their assignment to proteins. Furthermore, theoretical mass of proteins listed by databases and those measured often differ strongly, e.g., as shown for CM3 (reference mass without signal peptide 15.8 kDa and measured mass 15.5 kDa) by several studies (Capocchi et al., 2013; Call et al., 2019; Sagu et al., 2022).

The modern hexaploid common wheat is the most widely used wheat species for bread making and baked products worldwide. For pasta and other typical products of the Mediterranean diet, the tetraploid durum wheat is the dominant raw material. ATIs are present in all wheat species including durum and common wheat, spelt and emmer, but mostly not in diploid einkorn (Geisslitz et al., 2020), although Sielaff et al. (2021) have identified a trypsin inhibitor specific for einkorn. The distribution of individual ATIs differs between hexaploid (common wheat and spelt) and tetraploid (durum wheat and emmer) wheat species. The monomeric 0.19, the tetrameric CM1 and CM17, as well as the minor WCI are not present or in very low amounts in tetraploid wheat species. Furthermore, some durum wheat cultivars lack in monomeric 0.28 (Geisslitz et al., 2020; Sielaff et al., 2021). Proteins with amylase and/or trypsin inhibitory activity are encoded by 38 genes in the hexaploid common wheat cultivar “Chinese Spring” showing the high complexity of the genetic architecture of ATIs (Appels et al., 2018; Juhász et al., 2018; El Hassouni et al., 2021).

The analysis of proteins has the advantage in comparison to genomic-based methods that proteins may be not synthesized even though the genes are present, but not active or the mRNA is not stable. In the latter years, the development of novel sensitive and precise methods in combination with evaluation techniques has enhanced the analysis of proteins by proteomic-based methods. Indeed, the number of studies increased notably, which quantified ATIs in common wheat and other wheat species by targeted and untargeted liquid chromatography-tandem mass spectrometry (LC-MS/MS) (Henrottin et al., 2019; Bose et al., 2020; Geisslitz et al., 2020; Sagu et al., 2020; El Hassouni et al., 2021; Sielaff et al., 2021). These studies used different approaches, and studies comparing relative and absolute ATI quantitation approaches are very limited until now (Geisslitz et al., 2018; Sielaff et al., 2021). However, both approaches have advantages and disadvantages. The absolute quantitation requires expensive heavy isotopic labeled peptide or protein standards, but the stable isotope dilution analysis (SIDA) allows the precise and accurate absolute quantitation. However, results of different studies are hard to compare, since no validated ATI standard or reference material exists (Call et al., 2020; Geisslitz et al., 2020; Sielaff et al., 2021). Contrary to that, relative quantitation does not require peptide or protein standards resulting in a more cost-effective method, but with lower precision and no absolute statements are possible. Nevertheless, it is often sufficient to compare relative ATI abundancies within one sample set (Bose et al., 2020).

Gene silencing techniques including RNAi and CRISPR-Cas9 genome editing have the potential to reduce wheat immunoactivity. This already succeeded in common wheat by specific ATIs silencing by RNAi (0.28, CM3 and CM16) (Kalunke et al., 2020) and in durum wheat by CRISPR-Cas9 (CM3 and CM16) (Camerlengo et al., 2020). The silencing of genes may, however, not only affect the target genes, but may also change the expression of untargeted genes. This has been the case in RNAi silenced common wheat plants, in which high-molecular weight glutenin subunits (HMW-GS), important for the baking quality, were strongly silenced along with target proteins (Kalunke et al., 2020). On the other hand, durum wheat cultivar Svevo plants modified by genome editing showed silencing of only target genes CM3 and CM16, without any pleiotropic effects so far as shown by *in silico* analysis in coding regions (Camerlengo et al., 2020).

The aim of the present study was to compare different approaches to quantitate ATIs by LC-MS/MS. In order to prove the accuracy and evaluate performance of each method, absolute and relative quantitation of ATI concentrations and abundances in common and durum wheats, both wild type and silenced lines (as described above) were performed. In detail, the cultivars Bobwhite and Svevo and the corresponding silenced lines were analyzed by different quantitation approaches. On one hand, targeted LC-MS/MS and SIDA with a triple-quadrupole MS systems was performed. On the other hand, several untargeted approaches with data dependent acquisition (DDA) with and without labeling were performed on an IonTrap MS system and two Orbitrap MS systems. Last, data independent acquisition (DIA) on a QTOF system was tested.

## Materials and methods

### Wheat samples

Three transgenic lines (named 22-2, 24-1-1, and 10-10a) from the common wheat cultivar Bobwhite silenced by RNAi in the three ATI genes CM3, CM16, and 0.28 were obtained from a previous study of Kalunke et al. (2020). Durum wheat CRISPR-Cas9 edited lines in the ATI subunits CM3 and CM16 (R2P8c and R5P8b) were taken from the study of Camerlengo et al. (2020). Wholemeal flours (particle size ≤ 0.5 mm) were prepared by milling the grain samples with a laboratory Cyclone Mill (Cyclotec 1093, FOSS, Hilleröd, Sweden).

### Analysis of intact proteins

#### RP-HPLC-UV analysis of total extractable protein

Flour (50 mg) was extracted two times with 1 mL 50% 1-propanol in Tris-HCl (0.5 mol/L, pH 8.8) (v/v) containing 1% dithiothreitol (DTT) (w/v). For each extraction step, the samples were vortexed for 2 min and then stirred at 60°C for 30 min. After centrifugation at 3,750 × *g* and 22°C for 25 min, the supernatants were collected in a 2 mL flask and filled up with the extraction buffer. All extractions were made in triplicate. An aliquot of the supernatants (400 μl) were filtered (Whatman™, AQUA30/0.45 CA, GE Healthcare, Freiburg, Germany) and analyzed by reversed-phase high-performance liquid chromatography (RP-HPLC). A Prominence UFLC (Shimadzu, Kyoto, Japan) equipped with an Acclaim™ 300 C18 column (3 μm, 2 × 150 mm) was used. Solvent A was 0.1% trifluoroacetic acid (TFA) in water (v/v) and solvent B was 0.1% TFA in acetonitrile (ACN) (v/v). The flow rate was 0.4 mL/min and the column was tempered at 60°C. Following gradient was applied: 0–0.4 min 5% B, 0.4–0.5 min 5–30% B, 0.5–18.0 min 30–60% B, 18.0–18.1 min 60–90% B, 18.1–20.1 min 90% B, 20.1–20.2 min 90–5% B, 20.2–36.0 min 5% B. Detection was done by measuring the UV absorbance at 210 nm. For external calibration, reference gliadin (2.5 mg/mL) from the Prolamin Working Group (PWG-gliadin) (van Eckert et al., 2006) was dissolved in 60% ethanol in water (v/v) and analyzed with different injection volumes (5, 10, 15, and 20 μL). Control of the system and integration was performed with LabSolutions (Shimadzu).

#### RP-HPLC-UV analysis of Osborne fractions

Albumins/globulins, gliadins, and glutenins were extracted according to the modified Osborne fractionation (Wieser et al., 1998) and quantified as described in Section “RP-HPLC-UV analysis of total extractable protein” by RP-HPLC.

#### SDS-PAGE

Flour (20 mg) was incubated with sample buffer [1 mL, 293 mmol/L sucrose, 246 mmol/L Tris, 69 mmol/L sodium dodecyl sulfate (SDS), 0.5 mmol/L ethylenediaminetetraacetic acid (EDTA), 0.2 mmol/L Serva blue G250, 0.2 mmol/L phenol red, 0.1 mmol/L HCl, pH 8.5] overnight under reducing conditions (DTT, 50 mmol/L). The solutions were heated for 10 min at 60°C prior to use. The PageRuler Unstained Protein Ladder (Thermo Scientific, Bremen, Germany) covered a molecular weight range of 10–200 kDa with 14 proteins. A NuPAGE 4–12% Bis-Tris Protein Gel (1.0 mm, 10-well, Invitrogen, Carlsbad, CA, United States) and 2-(N-morpholino)ethanesulfonic acid (MES) running buffer (50 mmol/L MES, 50 mmol/L Tris, 3.5 mmol/L SDS, 1 mmol/L EDTA, pH 7.7) was used. Protein fixing, staining and gel destaining was performed as described in Lagrain et al. (2012). The gels were documented by a LAS-3000 (Fujifilm, Tokio, Japan) and the pictures were processed with ImageReader LAS-3000, version 2.1 (Fujifilm) and Advanced Image Data Analyzer (version 3.27.001, Raytest Isotopenmessgeräte GmbH, Straubenhardt, Germany).

#### Relative gene expression

Reverse Transcriptase-PCR (RT-PCR) followed by the amplification of the 0.28 target gene in durum wheat was carried out according to Camerlengo et al. (2020). Total RNA was extracted from immature caryopses of Svevo plants (a set grown in chamber in 2020 and a set grown in field in 2022) at 7, 14, or 15 and 25 or 28 days post anthesis (dpa). The 0.28 gene was amplified with the primers pair 0.28_83F (Camerlengo et al., 2020) and the actin gene was used as reference gene (cACT_F/ActR). Three biological replicates per line were used for the reaction.

### Absolute LC-MS/MS analysis

#### Sample preparation

Flour (50 mg) was extracted twice with ammonium bicarbonate (Ambic) solution (0.5 mL, 50 mmol/L, pH 7.8) for 30 min at 22°C (Ambic extracts). The suspensions were centrifuged for 25 min at 3,550 rcf and the supernatants were combined. The extracts were evaporated to dryness in a rotational vacuum concentrator and the tubes were stored at −20°C until further analysis. For the TEP extraction, ice-cold acetone (6 mL) was added to exactly 1.5 mL TEP extracts of Section RP-HPLC-UV analysis of total extractable protein and proteins were precipitated overnight at −20°C. After centrifugation at 3,750 × *g* and 4°C for 25 min, the supernatant was removed and the pellet was washed with ice-cold acetone (2 mL). The tubes were stored at −20°C until further analysis. Dried Ambic extracts and TEP pellets were dissolved in Tris-HCl (320 μl, 0.5 mol/L, pH 8.5) and 1-propanol (320 μl). A mixture of internal standards containing 19 ^15^N/^13^C labeled peptides (50 μl) was added as described in Geisslitz et al. (2020). Reduction was performed with tris(2-carboxyethyl)phosphine (TCEP, 50 μl, 0.05 mol/L TCEP in 0.5 mol/L Tris-HCl, pH 8.5) and shaking for 30 min at 60°C and alkylation with chloroacetic acid (CAA, 100 μl, 0.5 mol/L CAA in 0.5 mol/L Tris-HCl, pH 8.5) for 45 min at 37°C in the dark. The solvent was removed by evaporation to dryness. Tryptic hydrolysis (0.5 mL, 0.2 mg trypsin per mL, 0.04 mol/L urea in 0.1 mol/L Tris-HCl, pH 7.8) was performed for 18 h overnight at 37°C in the dark. The reaction was stopped by adding 2 μl TFA. The solution was diluted 1 + 1 with 0.5 mL of 0.1% formic acid (FA) and filtered (Whatman, AQUA30/0.45 CA).

#### Absolute quantitation by LC-MS/MS-TripleQuad-SRM

For calibration, peptides and internal standards were mixed in molar ratios n(P)/n(IS) between 9.1 and 0.1 (9 + 1, 4 + 1, 3 + 1, 1 + 1, 1 + 3, 1 + 4, and 1 + 9). Samples and calibration were analyzed exactly as described in Geisslitz et al. (2020) using an UltiMate 3000 HPLC system (Dionex, Idstein, Germany) coupled to a triple-stage quadrupole mass spectrometer (TSQ Vantage, ThermoFisher Scientific). Data evaluation was performed using Skyline (version 21.1.0.278, MacCoss Lab Software, University of Washington, Seattle, WA, United States) (MacLean et al., 2010).

### Relative LC-MS/MS analysis

The mass spectrometry proteomics data have been deposited to the ProteomeXchange Consortium via the PRIDE partner repository (Perez-Riverol et al., 2022).

#### Total extractable protein extraction for relative LC-MS/MS analysis

Flour (25 mg) was extracted two times with 1 mL 50% 1-propanol in Tris-HCl (0.5 mol/L, pH 8.8) (v/v) containing 1% dithiothreitol (DTT) (w/v) (Svevo: 2 × 1.3 mL and R5P8b: 2 × 2.1 mL; Bobwhite and 22-2, 2 × 2.8 mL). For each extraction step, the samples were vortexed for 2 min and then stirred at 60°C for 30 min. After centrifugation at 3,750 × *g* and 22°C for 25 min, the supernatants were collected in 15 mL tubes. The solutions were homogenized by shaking. Aliquots (150 μl containing 150 μg protein) were transferred in 1.5 mL tubes. Proteins were precipitated with ice-cold acetone (600 μl) overnight at −20°C. After centrifugation at 3,750 × *g* and 22°C for 25 min, the supernatant was removed and the pellet was washed with ice-cold acetone (200 μl). The tubes were stored at −20°C, shipped to Australia and Austria and again stored at −20°C until further analysis.

#### Label-free quantitation by LC-MS/MS-IonTrap

The protein pellets of the TEP extraction containing 150 μg protein were resuspended with 30 μl urea (8 mol/L) and Ambic (0.1 mol/L, pH 7.8), reduced with 30 μl DTT (15 mmol/L in Ambic), and alkylated with 30 μL iodoacetamide (55 mmol/L in Ambic). Prior to digestion, proteins were precipitated with cold acetone, after drying, the pellet was resuspended with Ambic and digested with trypsin (10 μl, 0.1 mg trypsin per mL in 1 mmol/L HCl). A Thermo UltiMate 3000 capillary-flow UHPLC system (ThermoFisher Scientific) was equipped with a C18 column (nanoEase M/Z HSS T3 Column, 100 Å, 1.8 μm, 300 μm × 150 mm, Waters, Milford, MA, United States). A gradient from 1% B [B: 80% ACN in water (v/v) with 0.1% FA] to 40% B in 50 min was applied, followed by a 10 min gradient from 40% B to 95% B that facilitates elution of large peptides, at a flow rate of 6 μl/min. Peptide detection was done with a Bruker amaZon Speed Ion Trap (Bruker, Billerica, MA, United States) in the enhanced resolution mode, DDA mode (=switching to MS/MS mode for eluting peaks). MS-scans were recorded (range: 350–1600 Da) and the eight highest peaks were selected for fragmentation. Peptides were identified with MASCOT MS/MS Ions Search using the UniProtKB Taxonomy: Viridiplantae (Green plants) (March 13, 2021). Peptides were manually identified and curated with DataAnaylsis 4.0 (Bruker) based on MS1 peak area of selected peptides.

#### Label-based quantitation by LC-MS/MS-iTRAQ

The protein pellets of the TEP extraction containing 150 μg protein was treated as described in Kalunke et al. (2020) and a 4-plex kit (SCIEX, Darmstadt, Germany) was used to label the peptides. Svevo (57 μg each) was iTRAQ labeled as 114 and 116 and R5P8b (57 μg each) as 115 and 117 in the durum wheat repetition. Bobwhite (48 μg each) was iTRAQ labeled as 114 and 116 and 22.2 (48 μg each) as 115 and 117 in the common wheat repetition. Before LC-MS/MS analysis, the pooled digests were fractionated by high pH HPLC on an 1100 HPLC system (Agilent, Santa Clara, CA, United States) using a Zorbax C18 column (5 μm, 2.1 × 150 mm, Agilent). Peptides were eluted with a linear gradient of 20 mmol/L ammonium formate and 2% ACN in water (v/v) to 20 mmol/L ammonium formate and 90% ACN in water (v/v) at 0.2 ml/min. The 12 fractions were analyzed by an UltiMate 3000 RSLC nanoflow system (Thermo Scientific) coupled to a Q Exactive HF mass spectrometer (Thermo Scientific). Peptides were loaded onto an Acclaim™ PepMap™ (100 Å, 2 μm, 0.075 × 150 mm, Thermo Scientific) and separated with a linear gradient of water and ACN containing 0.1% FA (v/v).

Obtained data were processed with PEAKS X Pro (version 10.6, Bioinformatics solutions Inc., Waterloo, ON, Canada). According to the PEAKS quantitation workflow, data refinement was performed with the default settings (correct precursor: Mass only; associate feature with chimera scan: Enabled; filter features: Charge between 2 and 8). *De novo* search followed with error tolerance of precursor mass of 15 ppm and fragment ion of 0.5 Da, trypsin as enzyme and carbamidomethylation and iTRAQ 4plex (K, N-term) as modification. For the database search, a FASTA file containing only the 13 ATIs of interests were created and used as database. In the quantitation module, the labels were specified as described above. Quantitation mass tolerance was set to 0.2 Da and false discovery rate (FDR) threshold to 1.0%. Spectrum filter settings were set to −10LgP ≥ 48.3, quality ≥ 0, reporter ion intensity ≥ 0E0, detected in at least 1 channel and protein filters to significance ≥ 0, fold change ≥ 1, significance method: PEAKSQ, has at least 0 unique peptide.

#### Label-free quantitation by LC-MS/MS-Orbitrap

The protein pellets were reduced and alkylated as described in Section “Sample preparation” and after evaporation to dryness, the residues were dissolved in Tris-HCl (0.5 mL, pH 7.8, 0.1 mol/L Tris-HCl, 0.04 mol/L urea). Tryptic hydrolysis (5 μg trypsin, 1:50 enzyme-substrate-ratio) was performed for 18 h overnight at 37°C in the dark. The reaction was stopped by adding 2 μl TFA. Purification was done using Discovery DSC-18 solid phase extraction columns (100 mg, Sigma-Aldrich, MO, United States) according to manufacturer instruction and peptides were eluted with 40% ACN containing 0.1% FA. The solvent was evaporated and the peptides were stored at −20°C until further analysis. An UltiMate 3000 RSLC nano system was coupled to a Q Exactive Plus Orbitrap (ThermoFisher Scientific, Waltham, MA, United States). The injection volume was 0.6 μl corresponding to 600 ng peptides. Using a flowrate of 8 μL/min of 0.1% FA in water, the peptides were loaded onto a trap column for 5 min. Subsequently, the peptides were separated on an analytical column (bioZen Peptide XB-C18, 2.6 μm, 250 μm × 0.075 mm, Phenomenex, Aschaffenburg, Germany) using a flow rate of 300 nL/min, 0.1% FA in water (v/v) as solvent A, 0.1% FA in ACN containing 5% water as solvent B and a gradient of 0–5 min 2% B, 5–6 min 2–5% B, 6–45 min 5–20% B, 45–60 min 20-33% B, 60–62 min 33–100% B, 62–65 min 100% B, 65–66 min 100–2% B, 66–80 min 2% B and a column temperature of 40°C. The eluate from the analytical column was sprayed *via* a Nanospray Flex Series ion source (ThermoFisher Scientific) into the MS at a source voltage of 1.9 kV, at a capillary temperature of 250°C and S-lens level of 60. The Q Exactive Plus was set to DDA in positive ion mode, automatically selecting the 15 most intense precursor ions from the preceding full MS1 spectrum with an isolation width of 2.0 *m/z* at 28% normalized collision energy and default charge state of 2 + . MS1 (360–1300 *m/z*) spectra were acquired in the Orbitrap using a resolution of 70,000 (at 200 *m/z*), an automatic gain control (AGC) target of 1e6 and a maximum injection time (IT) of 50 ms. MS2 spectra, selecting ions with charge 2+ to 7+, were acquired using a resolution of 17,500, an AGC target of 1e5, a maximum IT of 50 ms and a fixed first mass of 140 *m/z*. Dynamic exclusion was set to 15 s.

The raw data were directly used as input in MaxQuant (version 1.6.17.0) (Tyanova et al., 2016). For the first search, a large FASTA file containing all entries from the genus *Triticum* of the UniProtKB database (download December 10, 2020) was used and a FDR of 100% on peptide level was applied. Carbamidomethylation on cysteines was specified as fixed modification and trypsin as proteolytic enzyme with up to two allowed missed cleavage sites. Match-between runs (matching time window 0.7 min, alignment time window 20 min) was enabled, and the results were filtered for a minimal length of seven amino acids. From the identified proteins in the first search, a smaller database was built in UniProtKB. This FASTA file was used for the second search with 1% peptide and protein FDR and in addition to the before mentioned parameters, the iBAQ algorithm was applied. A total sum normalization of iBAQ protein intensities between samples was performed to correct for different total protein injection amounts.

The MaxQuant peptide library was loaded in Skyline (version 21.2.0.369) and the proteins and peptides listed in Supplementary Table 1 were added. The automated integrated peaks were checked manually and adjusted. The MS1 peak areas were exported and used for relative quantitation.

#### Label-free quantitation by LC-MS/MS-QTOF

The protein pellets were suspended with Rapigest solution (0.05%, 150 μl) and the proteins reduced by addition of DTT (50 μl, 50 mmol/L) for 20 min at 60°C. After cooling to room temperature, alkylation was conducted with iodoacetamide (50 μl, 100 mmol/L) in the dark for 30 min. Remaining iodoacetamide was removed by a second addition of DTT (20 μl, 50 mmol/L). For digestion, an aliquot (100 μl) was mixed with trypsin solution (20 μL, containing 1.25 μg trypsin in 50 mmol/L Ambic at pH 8.0) and incubated at 37°C overnight. All these steps, except alkylation were conducted with a ThermoMixer shaking. Digestion was stopped with TFA (10 μl, 5% in water) and SPE clean-up of tryptic peptides was performed with stage-tips (5 layers of SDB-XC reversed-phase Empore extraktions diks, 3M Deutschland GmbH, Neuss, Germany), were filled in 200 μl pipette tips. After equilibration with methanol, loading and washing (twice with 2.5% ACN in water containing 0.1% TFA) peptides were eluted with 60% ACN in water and 0.1% TFA (v/v). The eluate was evaporated to dryness and dissolved in 10% ACN in water containing 0.1% FA (v/v).

Digested peptides were separated on a Waters H-class UPLC system using a gradient with water (A) and 98% ACN in water (B) containing 0.1% FA (gradient with 8% B to 40% B in 40 min.) at a flow rate of 0.1 mL/min and 60°C with a CSH C18 column (1.7 μm, 150 × 1 mm, Waters). Detection was performed with a Xevo G2 XS QTOF mass spectrometer from Waters. DIA was performed in SONAR™ mode and a 10 Da window for quadrupole scanning (from 400 to 1200 *m/z*) was used. Fragmentation was performed by ramping collision energy from 20 to 40 V in the range of 350–1850 *m/z*. Every 180 s lockmass calibration was achieved with leucine enkephalin (Waters SKU: 186006013). Recorded SONAR-spectra were processed with ProteinLynx Global Server (PLGS, Waters) with minimum three respectively seven product ions for peptides and proteins and proteins were identified with an imported *Triticum aestivum* database from UniProtKB containing 472 entries (all reviewed entries plus one unreviewed for CM17) using 4% FDR as recommended by the manufacturer. Carbamidomethyl was set as fixed and oxidation as variable modification; max. one missed cleavages was allowed, peptide and MS/MS tolerance was calculated automatically by the software. By applying the expression workflow, EMRT and protein tables with normalized intensities for identified peptides (PLGS score above 5) assigned to ATIs were generated. Peptides of each ATI were checked manually for concordant ones and these peptides were removed afterward to ensure that relative quantitation was based exclusively on unique peptides. Additionally, only peptides found in at least two of three replicates were used for quantitation.

### Statistics

Figures were created using Origin Pro 2022 (OriginLab Corporation, Northampton, MA, United States). One-way analysis of variance (ANOVA) with Tukey’s *post hoc* test and *t*-tests were performed with a significance level of 0.05 using Origin Pro 2022.

## Results

### Characterization of intact proteins

#### Qualitative analysis by SDS-PAGE

**FIGURE 1:**
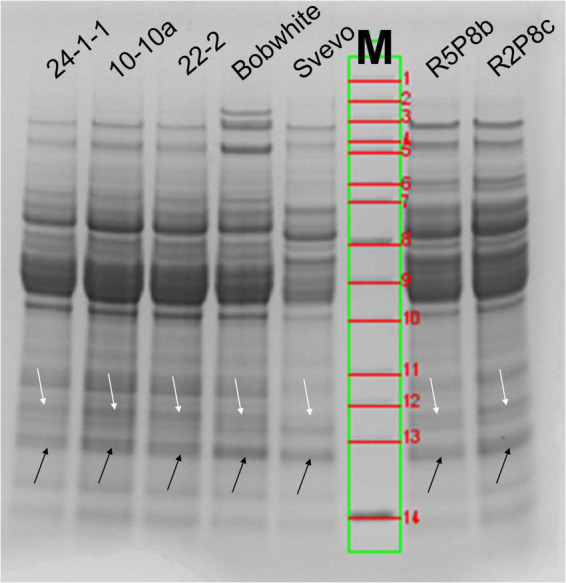
SDS-PAGE (4–12% Bis-Tris gel with MES-running buffer) of wheat extracts under reducing conditions (DTT). White arrows correspond to the band over 15 kDa (CM3) and black arrows below 15 kDa (all other ATIs). M, molecular weight marker: 1, 200 kDa; 2, 150 kDa; 3, 120 kDa; 4, 100 kDa; 5, 85 kDa; 6, 70 kDa; 7, 60 kDa; 8, 50 kDa; 9, 40 kDa; 10, 30 kDa; 11, 25 kDa; 12, 20 kDa; 13, 15 kDa; 14, 10 kDa.

SDS-PAGE is an easy and fast method for the preliminary characterization of wheat proteins. ATIs are especially present below and over 15 kDa (arrows in Figure 1). According to the calculated molecular weight of ATIs, the two faint protein bands over 15 kDa contains mainly CM3 in the wheat samples and the more intense protein band below 15 kDa all other ATIs. In Bobwhite, a sharp and intense band was visible below 15 kDa and two faint bands over 15 kDa. The three silenced hexaploid lines showed very similar patterns in this area of molecular weights. Overall, Svevo was characterized by fainter protein bands compared to the silenced tetraploid lines even though the same amount of flour was used for SDS-PAGE (not the same protein content, see Section “Quantitation of intact proteins”). Svevo also had two bands over 15 kDa, but the one with higher molecular weight was very faint. Furthermore, Svevo had a similar intense band below 15 kDa in comparison to Bobwhite. The silenced tetraploid lines showed very similar protein patterns compared to each other and to the hexaploid samples. All in all, it was not possible to confirm the silencing of the target ATIs by SDS-PAGE due to the insufficient resolution in the region of 11–18 kDa. However, the used combination of running buffer (MES) and gel (4–12% gradient Bis-Tris) is a promising system to use for in-gel digestion of ATIs.

#### Quantitation of intact proteins

For label-free and iTRAQ-LC-MS/MS analysis, it is crucial that all samples contain ideally the same amount of proteins to reach the best comparability between the samples. Thus, first, the protein content in the TEP extracts was analyzed by HPLC-UV to guarantee that each tube contained 150 μg protein for the subsequent LC-MS/MS analysis independently to the crude protein content of the kernels. Further, it was confirmed that the one step TEP protocol described in Kalunke et al. (2020) extracts comparable amounts of proteins in comparison to the well-established multi-step Osborne fractionation (Wieser, 1998).

**FIGURE 2:**
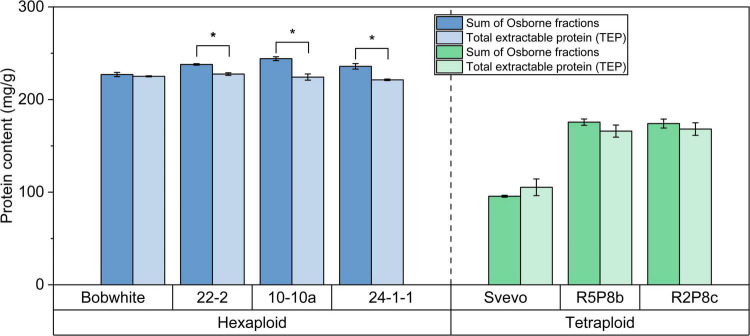
Sum of Osborne fractions (left bar) and total extractable protein (right bar) of hexaploid (blue) and tetraploid samples (green). *Significant difference between both extraction methods (*t*-test, *p* < 0.05).

The hexaploid Bobwhite and the silenced hexaploid lines were characterized by a very high content of TEP and sum of Osborne fractions between 221.3 mg/g and 244.2 mg/g (Figure 2). On the contrary, tetraploid Svevo showed a lower protein content for both extractions (Osborne: 95.6 mg/g; TEP: 105.3 mg/g). Both silenced tetraploid lines (166.0 mg/g and 175.6 mg/g) had almost doubled protein content in comparison to Svevo. With exception of Svevo, the Osborne fractionation resulted in an on average 5% higher protein content than the TEP extraction. This difference was significant for the three silenced hexaploid lines, but not for the other four samples (*t*-test, *p* < 0.05). Overall, both extraction methods revealed highly comparable protein contents.

### Absolute quantitation of amylase/trypsin inhibitors by stable isotope dilution assay

Twelve ATIs (0.19, 0.28, 0.53, CM1, CM2, CM3, CM16, CM17, WASI, CMX1/2/3, WCI, and WTI) were absolutely quantified by SIDA and LC-MS/MS according to Geisslitz et al. (2020). In this validated method, the ATIs were extracted with Ambic solution, which mostly extract ATIs, and very low amounts of gliadins and glutenins (Geisslitz et al., 2018). In contrast, in the TEP extraction, all wheat proteins are expected to be extracted.

**FIGURE 3:**
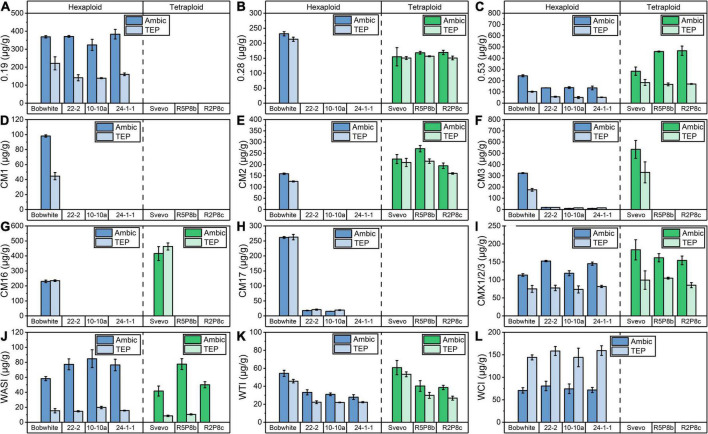
Quantitation of 12 ATIs by SIDA and LC-MS/MS after Ambic (left bar) and TEP (right bar) extraction in hexaploid (blue) and tetraploid samples (green): **(A)** 0.19; **(B)** 0.28; **(C)** 0.53; **(D)** CM1; **(E)** CM2; **(F)** CM3; **(G)** CM16; **(H)** CM17; **(I)** CMX1/2/3; **(J)** WASI; **(K)** WTI; **(L)** WCI.

In the hexaploid Bobwhite, all 12 ATIs were detectable (Figure 3), but in tetraploid Svevo, 0.19, CM17, WCI, and CM1 were not present, which is in agreement with previous studies (Geisslitz et al., 2020; Sielaff et al., 2021). The most abundant ATIs were 0.19 and CM3 in Bobwhite, which corresponded to about 30% based on all 12 ATIs. In Svevo, the most abundant ATIs were 0.53, CM2, CM3, and CM16, which corresponded to more than 75% based on all 12 ATIs. The Ambic and TEP extraction revealed comparable results for 0.28, CM2, CM16, CM17, and WTI, but remarkable differences were observed for the other ATIs. Overall, with exception of WCI, higher contents of individual ATIs were found in the Ambic extracts than in the TEP extracts. According to both extraction procedure, the silencing of 0.28 (Figure 3B), CM3 (Figure 3F), and CM16 (Figure 3G) succeeded for the silenced lines of Bobwhite and that of CM3 and CM16 for the silenced lines of Svevo.

As already described, the content of extractable proteins was similar in Bobwhite and the three silenced hexaploid lines and thus, changes in the absolute ATI content are comparable between Bobwhite and the silenced lines. The silenced lines of Bobwhite had a lower content of 0.53 (Figure 3C) and WTI (Figure 3K), whereas a higher content of CMX1/2/3 (Figure 3I) was detected. The silencing of the target ATIs 0.28, CM3, and CM16 led to very low amounts or to amounts lower than the limit of detection for CM1, CM2, and CM17. In contrast to that, the silencing of CM3 and CM16 did not lead to a complete absence of CM2 in the silenced tetraploid lines (Figure 3E). Nevertheless, CM2 was downregulated, if the doubled protein content of the silenced lines compared to Svevo is taken into account.

**FIGURE 4:**
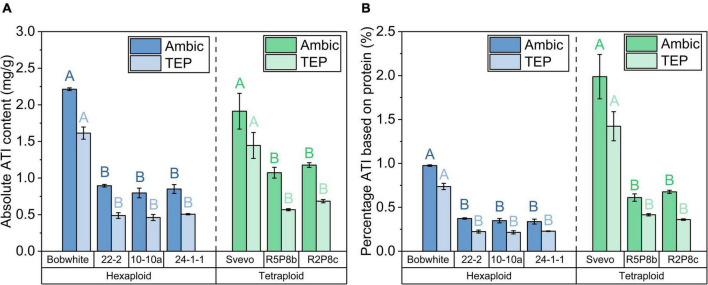
**(A)** Absolute ATI content as sum of 12 ATIs for Ambic (left bar) and TEP (right bar) extractions; **(B)** percentage ATI of Ambic extraction (left bar) based on the sum of Osborne fractions and that of TEP extraction (right bar) based on the quantitation of intact proteins in the TEP extraction for hexaploid (blue) and tetraploid samples (green). With exception to the two bars of Svevo in a, all left and right bars differed significantly (*t*-test, *p* < 0.05). The modifications differed significantly to Bobwhite and Svevo shown by different capital letters (one-way ANOVA with Tukey’s *post hoc* test, *p* < 0.05).

The sum of the 12 ATIs represents the absolute total ATI content. An absolute ATI content of 2.2 mg/g was observed in the Ambic extraction for Bobwhite and 1.9 mg/g for Svevo (Figure 4A). In agreement with individual ATIs, the TEP extraction revealed a lower total ATI content than the Ambic extraction. The silenced hexaploid lines showed on average a 60% lower total ATI content than Bobwhite and the silenced tetraploid lines a 40% lower one than Svevo. The reduction of total ATI content was even more pronounced when considering the absolute protein content (see Section “Quantitation of intact proteins”). Share of ATIs (considering the total protein content of each sample) was reduced on average for Ambic and for TEP extraction by about 70% in Bobwhite and in Svevo, respectively (Figure 4B).

### Relative quantitation of amylase/trypsin inhibitors

**FIGURE 5:**
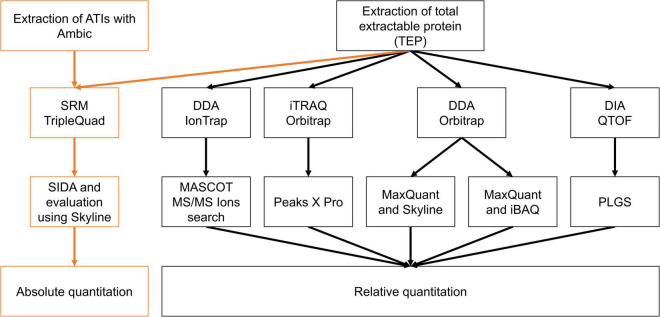
Overview of instruments, techniques, and evaluation procedures to compare the absolute and relative ATI content in the silenced samples compared to Bobwhite and Svevo.

For the relative quantitation, four different approaches were performed (Figure 5; Supplementary Table 2) and compared to the absolute quantitation using SIDA and LC-MS/MS. Relative quantitation was only performed from TEP extracts of Bobwhite, 22-2, Svevo and R5P8b, because the absolute ATI content of the hexaploid silenced samples 10-10a and 24-1-1 was comparable to 22-2 and that of R5P8b to R2P8c.

#### Identified and selected peptides for relative quantitation

A detailed list of all identified and selected peptides, which were used for relative quantitation, is included in Supplementary Table 1.

In the IonTrap analysis, the same peptides were used for the majority of ATIs (0.28, 0.53, CM1, CM2, CM3, and CM16) as the absolute quantitation used. Other peptides of these proteins were either identified with too low ion score or were even not detectable and thus, were not suitable for relative quantitation. For the two ATIs CM17 and WASI, other peptides had to be selected, because the peptides used in absolute quantitation were not detectable in the IonTrap experiment. For CMX1/2/3 one additional peptide was identified, for WCI, only one of the two peptides and the peptides of WTI were not identified. For 0.19, two other peptides than for the absolute quantitation was selected, because the peptides used in absolute quantitation were also present in 0.53 (see Section “Relative quantitation”). The overall number of peptides that were usable for quantitation were lower in the iTRAQ experiments than for the IonTrap analysis. In concordance to the IonTrap analysis, the peptides of WTI were not detectable. Furthermore, no peptides of 0.53 were identified. In contrast, the same peptide was detected for CM1 and CMX1/2/3. However, only one peptide was detected for CM2, CM3, WASI, WCI, respectively. Overall, for 0.28, 0.19, CM16, and CM17, other peptides were identified.

A higher number of peptides were detected in the DDA Orbitrap experiment compared to the IonTrap experiment, what was expected due to the use of a nanoLC-system and to superiority of the Orbitrap. With exception to one peptide of WTI, all identified peptides in the ATIs of interest are not unique (Supplementary Table 1) and thus, for iBAQ calculation not only unique peptides, but also razor peptides were enabled in MaxQuant. However, for the manual evaluation using Skyline, only those peptides were used for calculation, which were specific for one ATI-type. For example, only one peptide for either CM1 and CM2 was used, because the other two identified peptides were shared between both proteins. All in all, less peptides were used for the manual evaluation using Skyline in comparison to the iBAQ algorithm.

As the fourth alternative, the samples were analyzed by DIA. The selection of peptides was in high accordance with the absolute and the other relative quantitation methods for the majority of ATIs. Overall, the number of identified peptides was distinctly higher than for the Iontrap approach, whereas a few less peptides were found compared to the DDA Orbitrap experiment. The selection of peptides suitable to quantitate the specific ATIs worked well by PLGS except for 0.19 and 0.53. For these proteins, partly the same peptides were used, which were then manually excluded for quantitation. Finally, for the relative quantitation of 0.19, only two peptides, and for 0.53 only one peptide remained. A similar situation was observed for CM1 and CM2: For quantitation of CM1 only one peptide was selected, whereas CM2 was quantified by two peptides. Some peptides originating to CMX1/2/3 were identified, but not in all runs and thus excluded for quantitation. Contrary to the other relative approaches, peptides originating to WCI were not found. Instead, another chymotrypsin inhibitor (UniProtKB P82977) was identified by three significant matches (results not shown).

A few peptides containing methionine were selected. In general, such peptides should be avoided for quantitation due to variable oxidation of methionine, which can adulterate results. Nevertheless, for some proteins they are crucial because of their uniqueness, e.g., for 0.19 (SGPWMCYPGQAFQVPALPACRPLLR) and 0.53 (SGPWMCYPGQAFQVPALPGCRPLLK). This difficulty was evaded for absolute quantitation by using shared peptides for 0.19 (LQCNGSQVPEAVLR and LTAASITAVCR) and a unique one for 0.53 (EHGVSEGQAGTGAFPSCR), which allowed to calculate the amount of 0.19 by difference. However, this kind of solution cannot be applied to relative quantitation.

#### Relative quantitation

**FIGURE 6:**
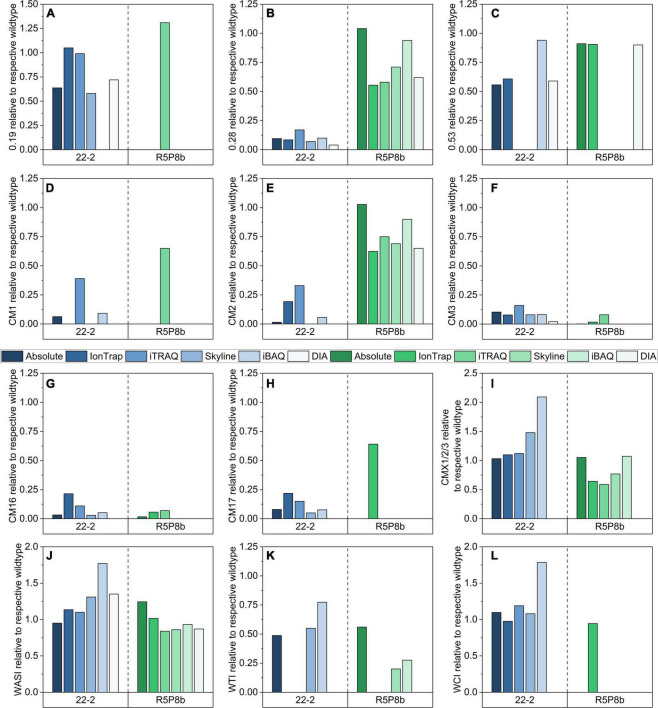
Relative comparison of 12 ATIs by means of absolute quantitation (SIDA), relative quantitation by IonTrap, iTRAQ, and Orbitrap using Skyline and iBAQ based on an individual selection of peptides for each approach: **(A)** 0.19; **(B)** 0.28; **(C)** 0.53; **(D)** CM1; **(E)** CM2; **(F)** CM3; **(G)** CM16; **(H)** CM17; **(I)** CMX1/2/3; **(J)** WASI; **(K)** WTI; **(L)** WCI.

Although the different methods used partially varying peptides for relative quantitation, the relative abundances for most ATIs generally agreed very well. However, some discrepancies were revealed as well (Figure 6). All five relative quantitation methods confirmed the reduction of CM3 (either not detected or between 0.02 and 0.08, Figure 6F) and CM16 (either not detected or between 0.06 and 0.07, Figure 6G) in the silenced tetraploid line. 0.28 (0.04-0.17, Figure 6B), CM3 (0.02-0.16), and CM16 (either not detected or between 0.03 and 0.21) were detected in distinct lower shares in the silenced hexaploid line.

Furthermore, all relative methods confirmed the downregulation of CM1 (either not detected or between 0.09 and 0.39, Figure 6D), CM2 (either not detected or between 0.06 and 0.33, Figure 6E), and CM17 (either not detected or between 0.05 and 0.22, Figure 6H) in the silenced line of Bobwhite. However, it was notable that the iTRAQ and IonTrap results revealed for these ATIs higher ratios than the absolute and the other relative methods.

The ATIs CM1, CM17, and WCI are generally not present in durum wheat. The majority of the relative methods revealed the same results as the absolute quantitation and detected no peptides for CM1, CM17, and WCI in Svevo. However, iTRAQ quantified CM1 in Svevo and in the silenced line as well (0.65). This result might be not correct due to a false positive identification, because in Bobwhite two peptides were identified for CM1, but in Svevo and the silenced tetraploid line, only one of these two peptides was found. Only the IonTrap experiment quantified CM17 (0.64) and WCI (0.95) in Svevo and its silenced line, but the other relative methods did not.

In contrast to the silenced line of Bobwhite, in which the most represented ATIs were downregulated (Figures 6B–H), the silenced line of Svevo had still a high content of the two ATIs 0.28 (Figure 6B) and CM2 (Figure 6E). The absolute quantitation revealed that Svevo and the silenced line contained almost the same amount (ratio of 1.00), but the relative quantitation methods revealed a slightly lower ratio between 0.55 and 0.94 for 0.28 and between 0.62 and 0.90 for CM2, respectively. All in all, iBAQ determined the highest ratios and was thus best comparable to the absolute quantitation. In contrast to the good performance of the iBAQ algorithm for 0.28 and CM2 in durum wheat, the iBAQ algorithm revealed 50–100% higher ratios for 0.53 (Figure 6C), CMX1/2/3 (Figure 6I), WCI (Figure 6L), WASI (Figure 6J), and WTI (Figure 6K) compared to the absolute and the other relative quantitation methods in Bobwhite. However, it should be added that with exception to WASI, not all methods were able to quantitate these ATIs in Bobwhite. Interestingly, the relative quantitation of CMX1/2/3 was not only dependent on the approach, but also on the wheat species. In Bobwhite, the IonTrap and iTRAQ results were very well comparable to the absolute quantitation, whereas the iBAQ and Skyline revealed higher ratios. Regarding Svevo, it was the other way around: The iBAQ experiment was best comparable to the absolute quantitation and the other methods revealed lower ratios.

**FIGURE 7:**
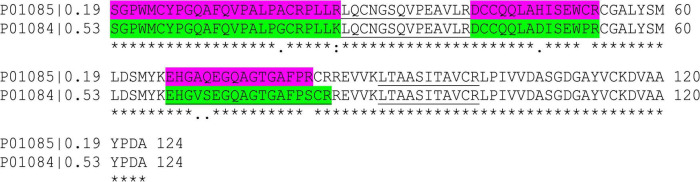
Amino acid sequence alignment of ATI 0.19 and 0.53 [performed with clustal omega, version 1.2.4. (Sievers and Higgins, 2018)]. The underlined peptides were used as internal standards in the SIDA experiment. The colored peptides differ between 0.19 and 0.53.

The largest discrepancies were revealed for the ATIs 0.19 (Figure 6A) and 0.53 (Figure 6C) between the different quantitation methods. The comparison of the amino acid sequences of both proteins (UniProtKB P01085 and P01084) by alignment using Clustal Omega (Sievers and Higgins, 2018) showed a very high homology (more than 94%). In detail, the two proteins differed only in three tryptic peptides (Figure 7). Applying absolute quantitation by SIDA, it was possible to distinguish between both proteins, because one of the three differing peptides of 0.53 (Supplementary Table 1) is used as internal standard to quantitate the difference between 0.19 and 0.53. In the applied absolute quantitation method, no unique peptide for 0.19 is currently present, but the quantitation of 0.19 by calculation of the difference was appropriate. In contrast, for relative quantitation, it is mandatory that only unique peptides are used. However, depending on the FASTA file used, the database contains several entries with very similar or even identical amino acid sequence. To circumvent this issue, either a very small database (as for the iTRAQ experiment) or an elaborate manual curation of the data (as done for IonTrap and Skyline, partially performed for PLGS) is required.

Nevertheless, different methods and algorithms still result in differing statements. The relative quantitation by IonTrap and DIA obtained very similar results for 0.53 between Bobwhite and Svevo and their silenced lines, as well as the absolute quantitation. In contrast, iTRAQ revealed that peptides of 0.53 were not present in any sample. The manual evaluation using Skyline found peptides of 0.53 only in Bobwhite and Svevo, but not in the silenced lines. Using the iBAQ algorithm, 0.53 was not detectable in Svevo and its silenced line. Furthermore, the relative quantitation of 0.53 revealed a two-times higher ratio compared to the absolute quantitation in Bobwhite and the silenced line. Summing up, it seems that the TEP extraction protocol had the biggest influence on the method performance regarding 0.53, since the differences between the relative methods were the highest. One reason for this might be the lower concentration due to the less effective extraction.

A higher accordance between the relative methods was revealed for 0.19 compared to 0.53, but not all methods obtained identical results. In Bobwhite, Skyline and DIA showed comparable results, but both IonTrap and iTRAQ revealed a two-times higher ratio. Furthermore, the iBAQ algorithm, evaluated an iBAQ intensity of 0 for Bobwhite and the silenced line. This is in high contrast to the manual evaluation by Skyline, since two of the three differing peptides assigned to 0.53 were detectable and quantitable in Bobwhite and the silenced line. With exception to iTRAQ, all relative quantitation methods revealed that 0.19 was not present in Svevo and its silenced lines.

### Expression of 0.28 in durum wheat

**FIGURE 8:**
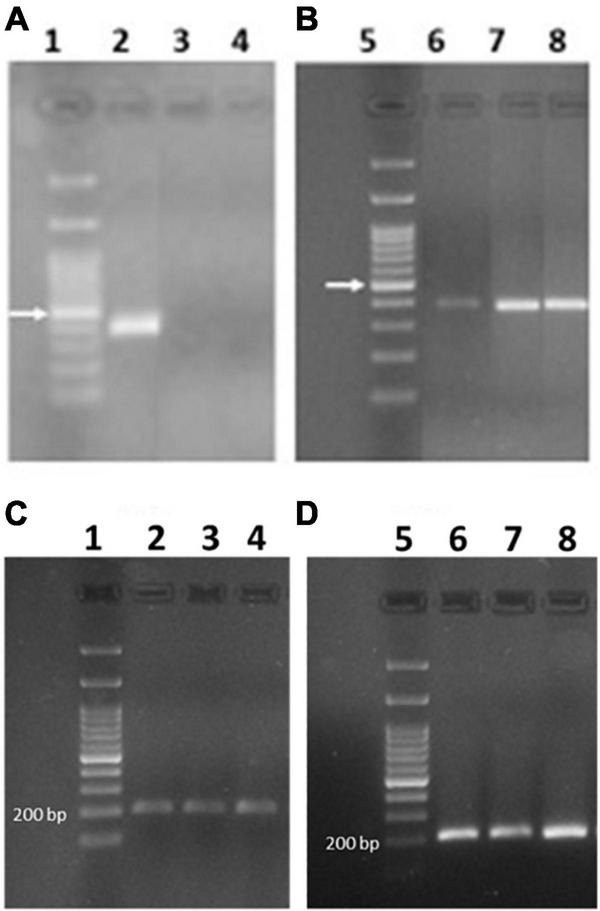
PCR amplicon electrophoresis of expressed 0.28 **(A,B)** and actin **(C,D)** genes. In lanes 1 and 5 the DNA ladder is reported and the arrow indicates the band at 500 bp. **(A,C)** Svevo grown in chamber at 7, 14, and 28 dpa (lanes 2–4, respectively). **(B,D)** Svevo grown in the field and collected at 7, 15, and 25 dpa (lanes 6–8, respectively).

Although Camerlengo et al. (2020) found expression of 0.28 gene only in the silenced lines and not in the Svevo control plants, 0.28 was detected in the tetraploid durum wheat Svevo using both absolute quantitation by SIDA and all relative quantitation approaches. Since Camerlengo et al. (2020) amplified the 0.28 gene from immature seeds at only one stage (14 dpa), the amplification of 0.28 transcripts was carried out also on cDNA obtained from Svevo plants at three different stages (7, 15, 25 dpa) to assess the expression of the active 0.28 gene during seed ripening. We used either the mRNA previously extracted from Svevo plants cultivated in growing chambers in 2020, and from a new set of Svevo plants grown in 2022 in the field. Surprisingly, we found contradictory results: Whereas by using mRNA from Svevo grown in growing chambers, we identified an amplification band corresponding to the 0.28 transcript in Svevo at 7 dpa, but not later (Figure 8A), by using mRNA from Svevo grown in the field, we always found an amplification band at all developmental stages, although very faint at 7 dpa (Figures 8B–D). This puzzling result might have different explanations: Either the expression of 0.28 is influenced by environmental factors (growing conditions were different), or the durum wheat cultivar Svevo shows heterogeneity, and the line used for plant transformation differs from the one used for field growing, although they do not differ phenotypically.

## Discussion

### Extractability of proteins

In the Osborne fractionation, proteins are extracted according to their solubility. First, both albumins and globulins are extracted by diluted salt solutions. Subsequently, gliadins are extracted with aqueous ethanol and last, glutenins under reducing conditions (e.g., with DTT) and high temperature (e.g., 60°C). This modified Osborne fractionation is time consuming and alternatives with lower number of extraction steps were established to extract all or the majority of proteins (van den Broeck et al., 2009; Henrottin et al., 2019; Jira and Münch, 2019). Furthermore, specific protocols were developed to extract ATIs (Geisslitz et al., 2018; Call et al., 2019; Sielaff et al., 2021; Sagu et al., 2022) using extractions solvents containing Ambic and/or NaCl with a pH between 7 and 8 and without any organic solvents and reducing reagents. We revealed that in our sample set, the Osborne fractionation and the TEP extraction resulted in comparable protein content by RP-HPLC-UV. Thus, the one-step TEP extraction is sufficient to extract the majority of all wheat proteins. However, the absolute ATI quantitation showed that the total ATI content was in the Ambic extracts distinct higher than in the TEP extracts. Some ATIs were more affected (e.g., 0.19, 0.53, and WASI) and had lower contents in the TEP extraction, some ATIs showed nearly the same content (e.g., 0.28, CM2, CM16, and WTI) and only WCI had a much higher content in the TEP extracts. Two possible reasons might be conceivable: First, the TEP extraction might be less effective to solubilize ATIs or second, the more complex sample containing higher amounts of proteins led to suppression in the MS analysis. The latter reason is unlikely in SIDA due to the use of heavy isotopic labeled standards, which are affected by suppression in an equal extent as the analyte. For barley ATIs, it was already demonstrated that the extraction solvent has an effect on the extraction efficiency (Bose et al., 2019). Depending on the ATI-type, the BDAI (similar to 0.19 and 0.53) was extracted best with both a buffer containing Tris-HCl and DTT and a buffer containing 55% 2-propanol and DTT, but CMa (similar to CM1) best with 55% 2-propanol and DTT. Furthermore, the BMAI (similar to 0.28) showed low extractability in the two aforementioned buffers, but best in a buffer containing urea, Tris-HCl and DTT. The study of Bose et al. (2019) and our study shows that the extraction is a crucial step in wheat proteomics. To avoid that the extraction had an influence on the different relative quantitation, the extraction was performed by a single person. The extracts were first divided in aliquots, then the proteins were precipitated and, last, the protein pellets were then shipped to the participating labs. To conclude, if the main study focus is only on the analysis of ATIs, aqueous extracts such as Sielaff et al. (2021) used are strongly recommended. However, if all wheat proteins are of interest, a suitable extraction solvent has to be chosen like the one for TEP used in this study. This was the reason to select an extraction procedure for all wheat proteins as well, because subsequent studies will focus on gluten proteins in the current sample set (unpublished).

### Peptides in other studies

Next to the extraction, the selection of peptides is a crucial step for accurate and precise quantitation. Depending on the used MS platform, ionization and detection characteristics of peptides varies leading to varying intensities and differing results between different MS methods. The ‘golden standard’ for protein quantitation by LC-MS/MS is the use of heavy isotopic labeled standards and SIDA (Ludwig and Aebersold, 2014). Even superior is the use of quantitation conCATamers (QconCAT), which reflects additionally the digestion (Brownridge et al., 2011). Both setups consider the effect of different ionization and detection properties, and the influence of matrix among others. For the development of these targeted methods, peptides must be chosen in advance. In contrast, for untargeted approaches, data is first acquired and afterward, peptides of interest are selected.

Comparing the selection of peptides for ATI quantitation with other studies, similarities but also differences are observable. In the study of Sielaff et al. (2021), label-free quantitation by DIA and absolute quantitation using QconCAT were performed by LC-MS/MS-QTOF in five different wheat species including common and durum wheat. All peptides except the two peptides for a 0.19-like ATI were among the ones selected for our study in the QconCAT standard. These two peptides were identified in the MaxQuant search of the LC-MS/MS-Orbitrap analysis and in the DIA experiments at the LC-MS/MS-QTOF by PLGS. Because the aim of the study was the comparison of the absolute quantitation by SIDA with different relative quantitation methods, no further detailed evaluation of this 0.19-like ATI was performed.

Bose et al. (2020) performed a discovery proteomics approach using a LC-MS/MS-QTOF system followed by a targeted LC-MS/MS-TripleQuad method to verify the diversity of ATI in common wheat by relative quantitation. Finally, 63 peptides representing 18 ATIs were selected for the targeted experiments. Overall, strong accordance with our study was seen, when proteins and peptides were compared. Nevertheless, Bose et al. (2019) were not able to differentiate between 0.19 and 0.53, but they used one peptide originating from 0.53 (not from 0.19) to measure a dimeric alpha-amylase inhibitor (UniProtKB C3VWC3). Furthermore, the trypsin inhibitor CMc (*Aegilops tauschii*, UniProtKB N1QTW5) was included with a peptide that we used to quantitate WCI in our study. Last, Bose et al. (2020) used two peptides of an uncharacterized protein (AAI domain-containing protein, UniProtKB A0A1D5UB33) and we used them for the WTI according to a study of Altenbach et al. (2011). This shows again the complexity of the UniProtKB database and the presence of redundant and multiple entries for very similar proteins.

Rogniaux et al. (2015) analyzed the relative abundance of some allergens including the ATIs 0.19, 0.28, CM1, CM2, and CM3 by a targeted approach (LC-MS/MS-Orbitrap). All the used peptides were found in several approaches of our study, except one peptide of CM2 that contained one missed cleavage. In accordance to our study, 0.19 and CM1 were absent in tetraploid wheat species.

The study of Sagu et al. (2020) applied an *in silico* approach to select potential peptides followed by LC-MS/MS-TripleQuad experiments to develop a comprehensive screening method for ATIs. Finally, 44 peptides were selected to relatively quantitate 15 ATIs. Comparison of peptides showed again high conformity. However, a few discrepancies were recognized: e.g., two other peptides were used to quantitate 0.28 and CM16, respectively. Aside from that, further proteins that were not analyzed in our targeted approach were monitored. In detail, a PUP88 protein (UniProtKB P93602), which is a member of trypsin/amylase inhibitors family from cereals, and an allergen C-C (UniProtKB P81496) protein were included.

Another study dealing with allergen detection used one peptide each for CM3 and 0.28 that were different from our study, to quantitate wheat gluten allergens by a targeted LC-MS/MS-TripleQuad method (Henrottin et al., 2019). Although we did not choose these peptides for the SIDA approach, these two peptides were considered by some relative quantitation experiments.

Summing up, the selection of peptides for the more abundant ATIs (0.19, 0.28, 0.53, CM2, CM3, and CM16) revealed high accordance with data found in literature compared to our study. However, for minor ATIs (CM1, CM17, CMX1/2/3, WASI, WTI, and WCI) partially alternative peptides were found. However, due to the high number of potentially unique or selective peptides for wheat ATIs, the individual selection for quantitation showed high variability. These circumstances can be partially explained by the huge number of entries for *T. aestivum* with redundant amino acid sequences, which is caused by homologous genes in the hexaploid genome (Bose et al., 2020; Sielaff et al., 2021). On the other hand, the setups of each mass spectrometry system affect the detection of single peptides, which might be causing divergences for choice of most suitable peptides for quantitation.

### Performance of different setups

In the latter years, quantitative MS-based proteomics technologies helped to answer important questions in plant biology. In general, chemical labeling-based workflows seem to have higher quantitative accuracy, but this approach has also some disadvantages like ratio distortion and sample interference as well. Due to the higher costs of labeling-based workflows, LFQ is one of the major techniques for comparative quantitative experiments to characterize the plant proteomes. So far, DDA among all kind of LC-MS/MS systems (Orbitrap, QTOF, IonTrap) was the main choice. Recently, DIA has shown a protein coverage that is equal to, or even exceeds, the one of DDA (Mehta et al., 2021). Due to its complex handling and analysis of DIA data, the evaluation is more laborious, but quantitation by DIA is more robust compared to DDA. The combination of targeted and untargeted (DDA) LC-MS/MS methods is recommended, when proteins should be quantified on a broad scale in an unbiased manner, while selecting specific proteins for quantitation (Hart-Smith et al., 2017).

In this study, a targeted approach was compared with untargeted ones, which included labeled iTRAQ and LFQ based on DDA and DIA as well. Furthermore, the data acquired at different MS instruments were evaluated with various software solutions. Summing up, rating and ranking of applied methods is challenging. However, a few conclusions can be drawn. As already intensively discussed, selection of peptides affects the results in a high degree, which is influenced strongly by the used software. Low resolution MS delivered a moderate performance. On the one hand, for most ATIs, proper results were achieved, but on the other side some ATIs were not or falsely quantified. LFQ with high resolution mass spectrometer revealed superior performance regarding identification and quantitation. Comparison between DDA and DIA is more or less unfeasible, because of different MS equipment, LC flow rates and software for quantitation. The high flow rate together with less sensitivity might be responsible that some proteins could not be measured by DIA. The labeled iTRAQ relative quantitation revealed an ambiguous performance, because the 0.53 was not detected, even though this ATI should be present, whereas the CM17 was quantified in the tetraploid Svevo, that should be actually not present. It might be conceivable that the false-positive identification of CM17 is due to the software and the missing identification of 0.53 due to the low concentration and the high-pH fractionation. Using SIDA together with high sensitive and selective TripleQuad MS instruments is for sure the golden standard for absolute quantitation, but showed strong limitations, if not only the target proteins are of interest. Furthermore, the high costs for the heavy isotopic labeled standards must be taken into account.

To conclude, all used approaches revealed mostly consistent results. However, some discrepancies were observed, especially regarding 0.19/0.53 and a few minor ATIs such as CM17. Although diverse setups were used, mainly the same peptides were assigned as suitable for quantitation, which was confirmed by other studies. Our findings raised the awareness that obtained results by proteomics software should be controlled regarding selection of unique or specific peptides. Often manual corrections are necessary to avoid false positive identification and incorrect or inaccurate quantitation. The high complexity of the wheat genome and high number of (almost) identical entries in databases are still a big challenge for quantitation of wheat ATIs. Furthermore, a distinct effect of extraction solvent was notable. If quantitation of ATIs is the main focus, the usage of salt solutions without organic solvents could be beneficial. By considering the mentioned circumstances ATIs can be quantified in wheat with high accuracy by LC-MS/MS.

## Data availability statement

The datasets presented in this study can be found in online repositories. The raw data and Skyline document of the absolute quantitation and of the relative quantitation using Skyline can be found in PanoramaWeb (https://panoramaweb.org/cVzu2W.url). The other mass spectrometry proteomics data have been deposited to the ProteomeXchange Consortium via the PRIDE (Perez-Riverol et al., 2022) partner repository with following dataset identifiers: PXD034458, PXD034478, PXD034769, and PXD034509.

## Author contributions

SG coordinated the extraction of proteins (TEP and Ambic), performed and evaluated the targeted experiments (SIDA followed by LC-MS/MS-TripleQuad-SRM) and relative quantitation without labeling on LC-MS/MS-Orbitrap (iBAQ and Skyline), involved in conceptualization of this study, and wrote the main part of manuscript. LB conducted and evaluated the SDS-PAGE and RP-HPLC-UV analysis. SI was responsible for relative quantitation by LC-MS/MS-iTRAQ and assisted in data analysis. CG-G performed the analysis by LC-MS/MS-IonTrap and the data analysis of measured spectra. FS and FC were responsible for relative gene expression experiment and its execution and developed the samples. SM supervised the gene expression experiment and was involved in conceptualization of the study. SD’A performed and evaluated the LC-MS/MS-QTOF (DIA) measurements, supervised the whole study, and wrote several parts of the manuscript. All authors read and revised the manuscript and approved the submitted version.
