# Supplementary material for: Absolute and relative quantitation of amylase/trypsin-inhibitors by LC-MS/MS from wheat lines obtained by CRISPR-Cas9 and RNAi
Source: Front Plant Sci. 2022 Aug 29;13:974881. doi: 10.3389/fpls.2022.974881 (PMC9465248; doi:10.3389/fpls.2022.974881)
Supplement: Supplementary file 1 [file Table_1.DOCX]

Supplementary Material

# Supplementary Tables (uploaded as excel file)

## Supplementary Table 1: Identified and selected peptides including results of Blast search

## Supplementary Table 2: Overview of used setups for sample preparation, analysis of tryptic peptides by LC-MS/MS, identification and quantification

## Supplementary Table 3: Description of figure captions

## Supplementary Table 4: Data of Fig. 2 including t-test

## Supplementary Table 5: Data of Fig. 3

## Supplementary Table 6: Data of Fig. 4 including ANOVA

## Supplementary Table 7: Data of Fig. 6
